# The prevalence of obstructive sleep apnea in mild cognitive impairment: a systematic review

**DOI:** 10.1186/s12883-019-1422-3

**Published:** 2019-08-15

**Authors:** Talha Mubashir, Lusine Abrahamyan, Ayan Niazi, Deween Piyasena, Abdul A. Arif, Jean Wong, Ricardo S. Osorio, Clodagh M. Ryan, Frances Chung

**Affiliations:** 1 Department of Anesthesia and Pain Medicine, University Health Network, University of Toronto, MCL 2-405, 399 Bathurst Rd., Toronto, ON M5T2S8 Canada; 2 Institute of Health Policy, Management and Evaluation (IHPME), University of Toronto, Toronto, ON Canada; 3 Toronto Health Economics and Technology Assessment (THETA) Collaborative, Toronto General Hospital Research Institute, University Health Network, Toronto, ON Canada; 4 Department of Biology, University of Trent, Peterborough, ON Canada; 5 Department of Health Sciences, University of McMaster, Hamilton, ON Canada; 6 Department of Life Sciences, University of Toronto, Toronto, ON Canada; 7 Department of Psychiatry, NYU School of Medicine, New York, NY USA; 8 Centre of Sleep Health and Research, Department of Medicine, University Health Network, University of Toronto, Toronto, Ontario Canada

**Keywords:** Obstructive sleep apnea, Mild cognitive impairment, Prevalence

## Abstract

**Background:**

Previous studies have shown that obstructive sleep apnea (OSA) is associated with a higher risk of cognitive impairment or dementia in the elderly, leading to deleterious health effects and decreasing quality of life. This systematic review aims to determine the prevalence of OSA in patients with mild cognitive impairment (MCI) and examine whether an association between OSA and MCI exists.

**Methods:**

We searched Medline, PubMed, Embase, Cochrane Central, Cochrane Database of Systematic Reviews, PsychINFO, Scopus, the Web of Science, ClinicalTrials.gov and the International Clinical Trials Registry Platform for published and unpublished studies. We included studies in adults with a diagnosis of MCI that reported on the prevalence of OSA. Two independent reviewers performed the abstract and full-text screening, data extraction and the study quality critical appraisal.

**Results:**

Five studies were included in the systematic review. Overall, OSA prevalence rates in patients with MCI varied between 11 and 71% and were influenced by OSA diagnostic methods and patient recruitment locations (community or clinic based). Among studies using the following OSA diagnostic measures– self-report, Home Sleep Apnea Testing, Berlin Questionnaire and polysomnography– the OSA prevalence rates in MCI were 11, 27, 59 and 71%, respectively. In a community-based sample, the prevalence of OSA in patients with and without MCI was 27 and 26%, respectively.

**Conclusions:**

Based on limited evidence, the prevalence of OSA in patients with MCI is 27% and varies based upon OSA diagnostic methods and patient recruitment locations. Our findings provide an important framework for future studies to prospectively investigate the association between OSA and MCI among larger community-based cohorts and implement a standardized approach to diagnose OSA in memory clinics.

**PROSPERO registration:**

CRD42018096577.

**Electronic supplementary material:**

The online version of this article (10.1186/s12883-019-1422-3) contains supplementary material, which is available to authorized users.

## Backgroud

Mild cognitive impairment (MCI) and obstructive sleep apnea (OSA) are chronic, debilitating disorders that commonly occur in older individuals and may share a common pathological link [1]. The epidemiology of OSA and MCI is poorly understood and very few studies have assessed their relationship [1, 2].

Different diagnostic criteria for MCI have been proposed over time [3]. With the current criteria [4–9], the prevalence of global MCI in individuals aged ≥60 years is reported to be between 6 and 20% [10, 11] with rates being affected by several modifiable [12] and non-modifiable [12–15] factors.

OSA is a recurrent obstruction of the upper airway during sleep that leads to intermittent hypoxia, high intrathoracic pressure swings and sleep fragmentation that has recently been shown to be associated with a higher risk of MCI or dementia in elderly [1, 16, 17]. Untreated OSA in middle-age causes impairments in attention, vigilance, some aspects of memory, psychomotor performances and executive function [16, 18–20]. Furthermore, associations between OSA and cognition in middle-age and late-life are highly variable and the findings differ based on the definition of apnea hypopnea index (AHI) and setting of the study (clinic vs community). There is evidence suggesting that intermittent hypoxia, which contributes to subsequent oxidative stress and endothelial dysfunction, could be a significant mediator in the deleterious effects of OSA on neurocognitive function [21], but the mechanism(s) involved in this association and the role of sleep fragmentation are unknown [22].

To date, the prevalence of diagnosed or undiagnosed OSA in the MCI population remains unknown. The objectives of this review were to determine the prevalence of OSA in patients with MCI and examine whether an association between OSA and MCI exists. Considering aging of the general population and the increasing prevalence of MCI and dementia, reliably estimating the prevalence of OSA in patients with MCI may guide future health resource planning to diagnose and treat OSA early in the elderly population [13].

## Methods

### Study design and registration

The protocol of this study was registered in the International Prospective Register of Systematic Reviews (PROSPERO) (CRD42018096577). We followed the Preferred Reporting Items for Systematic Reviews and Meta-analyses (Additional file 1: PRISMA) guideline [23].

### Inclusion criteria and outcomes

All studies on adults (age > 18 years) that reported the prevalence of OSA (primary outcome of interest) among patients with MCI using established diagnostic methods were included. In particular, the diagnosis of OSA should have been established using sleep studies such as type 1 laboratory polysomnography (PSG), or types 2–4 portable sleep monitors, or sleep questionnaires, or a physician diagnosis. The secondary outcome was the risk of OSA among MCI patients relative to the control population without MCI. From herein we will refer to this population as “controls”. We considered experimental, cohort, cross-sectional and case-control studies and excluded case reports, case series and commentaries. In addition, studies with a mixed population with neurodegenerative disorders such as dementia and defined sleep disorders other than OSA (e.g., central apneas) were also excluded. Only English language articles and human studies were included.

### Information sources and search strategies

With the help of an information specialist (ME), we conducted a comprehensive search for published and unpublished literature in the following electronic databases: Medline (Ovid), PubMed (non-Medline records only), Embase, Cochrane Central Register of Controlled Trials, Cochrane Database of Systematic Reviews, PsychINFO, Scopus (Elsevier), the Web of Science, ClinicalTrials.gov and the WHO International Clinical Trials Registry Platform. The electronic searches were conducted from the date of inception of the databases until May 1, 2018. The search strategy combined MeSH terms and keywords related to OSA with those related to MCI (see Additional file 2 for the search strategy in Medline). In addition, we hand searched the reference list of included full-text articles and review articles to capture studies potentially missed from the original search. The identified citations were imported into an EndNote database and duplicate records were removed.

### Study selection and data extraction

Two reviewers (AN and DP) independently screened the titles and abstracts of all studies that resulted from the search to determine eligibility for full-text screening. From these full-text articles, studies were included in the systematic review if the primary outcome was reported. A standardized data extraction list in Excel was used to collect information on study characteristics, participants’ characteristics, details on outcomes and on study quality. Data was extracted from eligible full-text articles independently by two reviewers. Disagreements were resolved by the senior author (FC). When relevant, study authors [2, 24] were contacted for clarification and provision of additional information for the systematic review.

### Assessment of study quality

Two reviewers (AN and DP) critically appraised each included study by using the Joanna Briggs Institute critical appraisal checklist for analytical cross-sectional studies [25]. The checklist included the following 7 items: (1) appropriate recruitment of participants; (2) representative sample of the target population; (3) use of objective, standard criteria for ascertaining the exposure (MCI) and the (4) outcome (OSA); (5) identifying and (6) adjusting for confounding factors; and [7] appropriateness of statistical analysis. Items were evaluated using ‘yes’/‘no’/‘unclear’ or ‘not applicable’ options.

## Results

### Search results

**Fig. 1:**
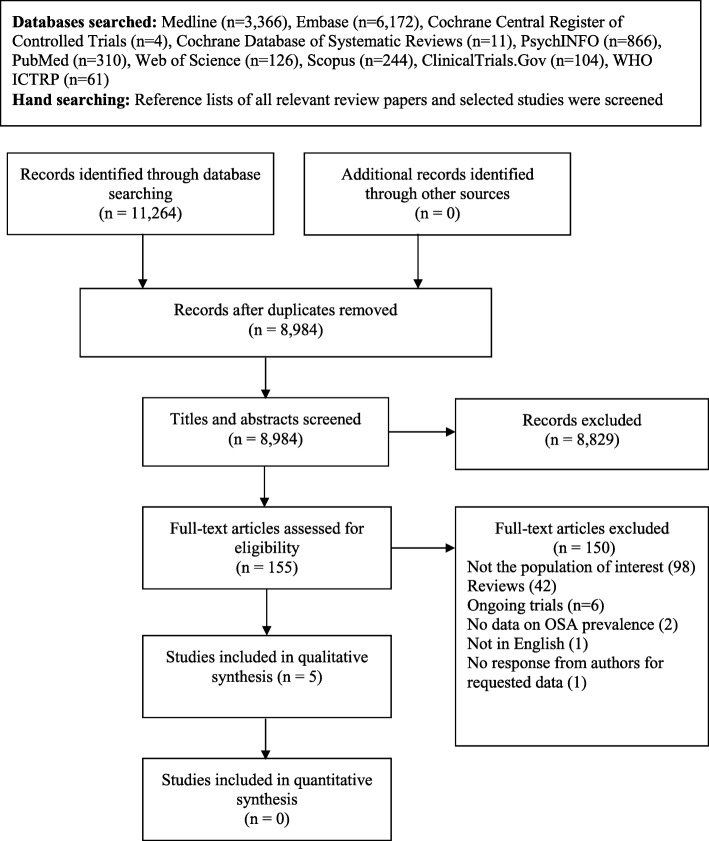
Flow diagram of study selection process. Abbreviations: WHO ICTRP = World Health Organization International Clinical Trials Registry Platform

The search returned 11,264 records in total. Of the 155 studies retrieved for full text review, 150 were excluded (Fig. 1). The most common reason for exclusion was having a different study population (*n* = 98). Five articles were included in the final systematic review [2, 24, 26–28].

### Characteristics of selected studies

The characteristics of the included studies are summarized in Table 1. Four studies [24, 26–28] had a cross-sectional design, while one [2] was a retrospective cohort. The studies were conducted in six different countries (Australia, Germany, Italy, South Korea, USA and Canada). The referral population of the five studies included elderly patients, with and without MCI, that were recruited from multiple clinics, including neurology [2, 26, 28] and general practitioner clinics (otherwise called Public Health Centers that are free clinics in South Korea to provide care to the lower socioeconomic classes and consists mostly of elderly patients) [27]. Only one study enrolled a randomly sampled, community-based general population [24]. A control population was included in four studies [2, 24, 27, 28], allowing for between-group comparison.

**Table 1:** Characteristics of studies included in the final review

Variables	Groups	Dlugaj et al. (2014) [24]	Guarnieri et al. (2012) [26]	Kim et al. (2011) [27]	Osorio et al. (2015) [2]	Wilson et al. (2014) [28]
Referral Population	–	General population (HNR cohort); age 45–75 yrs.	Neurology clinic; age > 60 years	General practitioner clinic (Public health centre); age > 60 years	Multiple clinics (ADNI cohort); age 55–90 yrs.	Neurology clinic; age > 50 years
Study design	–	Cross-sectional	Cross-sectional	Cross-sectional	Retrospective Cohort^a^	Cross-sectional
Study country	–	Germany	Italy	South Korea	USA and Canada	Australia
MCI diagnosis criteria	–	Petersen (2004) [6] Winblad (2004) [8]	Winblad (2004) [8]	Petersen (1999) [4]	Petersen (2005) [7]	Petersen (2005) [7]
OSA diagnosis method	–	ApneaLink™	History of snoring or sleep apneas & high risk on Berlin Questionnaire	PSG	“Patient reported OSA, followed by physician assessment of diagnosis based on patients’ medical history”	PSG
OSA diagnosis criteria	–	AHI ≥ 15 events/hour	NA	AHI ≥ 5 events/hour	NR	AHI ≥ 5 events/hour
OSA AHI Indices^b^	–	A: ≥80%, ≥10s H: 50–80%, ≥10s OD: NR	NA	A: ≥100%, ≥10s H: ≥50%, ≥10s OD: 3% (or arousal)	NR	A: ≥100%, ≥10s H: ≥50%, ≥10s OD: 3% (or arousal)
Number of subjects	MCI	230	138	30	402	37
	aMCI	120	NR	NR	402	10
	naMCI	110			0	27
	Controls	676	–	30	607	37
Number of males, n (%)	MCI	919 (51%)	50%	9 (30%)	–	NR
	Controls		–	9 (30%)		
Age (years), mean ± SD	MCI	63.79 ± 7.45	73 ± 9^c^	67.97 ± 4.09	–	65.5 ± 9.0
	Controls		–	67.37 ± 3.75		63.5 ± 8.7
BMI (kg/m^2^), mean ± SD	MCI	28.06 ± 4.38	NR	24.40 ± 3.28	–	27.6 ± 5.5
	Controls			24.49 ± 2.75		27.1 ± 4
Comorbidities (major)	–	DM (18%), HTN (36%), CAD (7%)	NR	NR	–	NR
Education (years), n (%) or mean ± SD	MCI	≤10: 167 (9%); 11–13: 1002 (56%); ≥14: 624 (35%)	8.2 ± 4.2^c^	6.80 ± 4.67	–	NR
	Controls		–	7.90 ± 5.11	–	NR
AHI (events/hour)	MCI	11.5 ± 11.43	NR	13.41 ± 11.61	NR	16.4 ± 16^d^
	Controls			15 ± 13.56		11.9 ± 10^d^
APOE positive (%)	MCI	445 (25%)	NR	NR	–	NR
	Controls					
Mini Mental Status Exam, mean ± SD	MCI	NR	27 ± 2^c^	NR	NR	28.1 ± 1.5
	Controls		–			29.2 ± 1.1

*Abbreviations: A* apnea, *ADNI* Alzheimer’s disease Neuroimaging Initiative, *AHI* apnea-hypopnea index, *APOE* Apolipoprotein E, *BMI* body mass index, *CAD* coronary artery disease, *DM* diabetes mellitus, *HNR* Heinz Nixdorf Recall, *HTN* hypertension, *H* hypopnea, *MCI* mild cognitive impairment, *NA* not applicable, *NR* not reported, *OD* oxygen desaturation, *OSA* obstructive sleep apnea, *PSG* polysomnography, *SD* standard deviation

^a^Data to calculate prevalence and/or odds ratio were provided by the study authors and were taken from baseline measurements of OSA and MCI (cross-sectional)

^b^The percentage drop of airflow from baseline for 10 s or more with or without oxygen desaturation

^c^Values estimated from a bar-graph

^d^AHI data was available on 24 out of the 37 subjects with MCI and 25 out of the 37 control subjects

The total number of patients with MCI and controls were 837 (range: 30–402) and 1353 (range: 30–676), respectively. The majority of MCI patients, with reported information on the type of MCI, had amnestic MCI (532 aMCI vs. 137 non-amnestic (na)MCI). Whether these individuals had impairments in a single or multiple cognitive domains was not reported. The mean age of patients ranged from 63.8 (CI:63.4–64.1) to 73 (CI:71.5–74.5) years and mean body mass index (BMI) from 24.4 (CI:23.2–25.6) to 28.1 (CI:27.9–28.3) kg/m^2^.

### MCI criteria

The inclusion criteria utilized by the studies for diagnosing MCI were largely similar [4, 6–8]. The diagnosis of MCI was made if the patient met the following criteria: 1) cognitive complaint from either the participant and/or family member, 2) objective cognitive impairment not normal for age, 3) preserved activities of daily living and, 4) absence of dementia (does not meet criteria for a dementia syndrome). Participants that met criteria 3 and 4 and had subjective and objective memory complaints were categorized as having aMCI, while those with deficits in cognitive domains other than memory (e.g. language, executive function etc.) were diagnosed with naMCI [24, 28].

### OSA criteria

The OSA diagnostic method differed across the selected studies. To diagnose OSA, two studies utilized PSG [27, 28], one used the ApneaLink™ [24] (a portable sleep apnea testing device), one used the Berlin Questionnaire [26], and another used patients’ “self-reported information followed by physician assessment based on patients medical history” [2], which will be referred to “self-report” from herein. The two studies that used PSG considered patients having OSA if they had an AHI ≥ 5 events/hour. The study that used the ApneaLink [24] device, applied an AHI cut-off of ≥15 events/hour to diagnose OSA.

### Quality of studies

The critical appraisal of the identified studies is presented in Table 2. All studies defined the inclusion criteria and described the study population in sufficient details, providing references to the original study and cohort from which participants were recruited when relevant. An objective and standard criteria was used to diagnose the exposure/condition, MCI, and appropriate statistical analysis was used. Although confounding factors such as age, sex, type of MCI, level of education or the presence of *APOE* gene were identified, they were not dealt with in the data analysis for all studies. Finally, only two studies [27, 28] used the current gold standard, PSG, to measure the outcome, OSA.

**Table 2:** Quality of included studies

Author	Criteria for inclusion in the sample clearly defined?	Study subjects and the setting described in detail?	Exposure measured in a valid and reliable way?	An objective, standard criteria used for measurement of the condition?	Confounding factors identified?	Strategies to deal with confounding factors stated?	Outcomes measured in a valid and reliable way?	Appropriate statistical analysis used?
Dlugaj et al. [24]	Yes	Yes	Yes	Yes	Yes	Yes	No	Yes
Guarnieri et al. [26]	Yes	Yes	Yes	Yes	Yes	Yes	No	Yes
Kim et al. [27]	Yes	Yes	Yes	Yes	Yes	No	Yes	Yes
Osorio et al. [2]	Yes	Yes	Yes	Yes	Yes	Yes	No	Yes
Wilson et al. [28]	Yes	Yes	Yes	Yes	Yes	No	Yes	Yes

### Prevalence of OSA in MCI

**Fig. 2:**
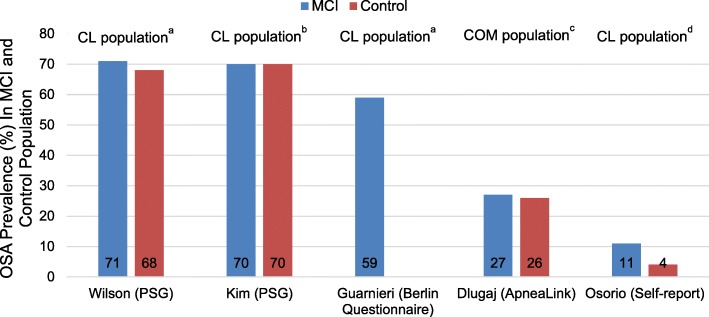
Reported OSA prevalence (%) in patients with MCI and Controls. Abbreviations: CL = clinic; COM = community; HNR = Heinz Nixdorf Recall; MCI = mild cognitive impairment; PSG = polysomnography. ^a^Includes patients recruited from neurology clinics. ^b^Includes patients recruited from a public health center. ^c^Includes patients recruited from HNR cohort (community-based sample). ^d^Includes patients recruited from multiple clinics, including neurology clinics, OSA risk in MCI vs. Controls [OR 3.61 (2.09–6.22), *p* <  0.0001]

Five studies documented the prevalence of OSA in individuals with MCI, of which four included the prevalence of OSA in the control population (Table 3). Overall, results indicated that OSA is present in 11–71% of MCI population compared to 4–70% in controls (Fig. 2). The prevalence of OSA in MCI was the highest among two studies that used PSG to diagnose OSA and recruited elderly patients from a clinic-based sample, 70 and 71%, respectively [27, 28]. In a community-based sample population, the prevalence of OSA in patients with and without MCI was 27 and 26%, respectively [24]. For studies using the following OSA diagnostic measures– self-report [2], ApneaLink [24] and Berlin Questionnaire [26]– the OSA prevalence rates in MCI were 11, 27 and 59%, respectively.

**Table 3:** The reported prevalence and odds ratio of OSA in MCI population in included studies

Author	Groups	Total sample	Subjects with OSA, n	Prevalence % (95% CI)	Odds Ratio (95% CI)	*P*-value
Dlugaj et al. [24]	MCI	230	61	27 (21.0–32.8)	1.03 (0.74–1.45)	0.84
	aMCI	120	32	52 (39.4–65.2)		
	naMCI	110	29	48 (34.8–60.6)		
	Controls	676	174^a^	26 (22.5–29.2)		
Guarnieri et al. [26]	MCI	138	81	59 (50.0–66.9)	–	–
	aMCI	NR	NR	–		
	naMCI					
	Controls	–	–	–		
Kim et al. [27]	MCI	30	21	70 (50.4–84.6)	1.00 (0.33–3.02)	1.00
	aMCI	NR	NR	–		
	naMCI					
	Controls	30	21	70 (50.4–84.6)		
^a^Osorio et al. [2]	MCI	402	44	11 (8.2–14.5)	3.61 (2.09–6.22)	< 0.0001
	aMCI	402	44	100 (90–100)		
	naMCI	–	–	–		
	Controls	607	23	4 (2.5–5.7)		
Wilson et al. [28]	MCI	37	17/24^b^	71 (48.8–86.6)	1.14 (0.34–3.86)	0.83
	aMCI	10	NR	–		
	naMCI	27				
	Controls	37	17/25^b^	68 (46.5–84.3)		

*Abbreviations: CI* confidence interval, *MCI* mild cognitive impairment, *aMCI* amnestic mild cognitive impairment, *naMCI* non-amnestic mild cognitive impairment, *OSA* obstructive sleep apnea

^a^Data provided by study authors

^b^AHI data was available on 24 out of the 37 subjects with MCI and 25 out of the 37 control subjects

### Risk of OSA in MCI

The risk of OSA in patients with MCI among included studies is summarized in Table 3. The risk of OSA in patients with MCI was over 3-fold compared to the control group in one study that recruited patients from multiple memory clinics (OR:3.61; 95% CI:2.09–6.22; *p*-value< 0.0001) [2]. There were no differences in risk of OSA between the MCI and control group in the rest of included studies with a control population [24, 27, 28].

## Discussion

### Summary of main results

To our knowledge, this study is the first systematic review evaluating the prevalence and risk of OSA in patients with MCI. In total, we found five studies reporting the prevalence of OSA in MCI showing considerable variations (11–71%). Two studies that used PSG [27, 28] to diagnose OSA showed that the prevalence of OSA in patients with MCI is high, 70%, while there is no significant association between having OSA and MCI. Furthermore, the prevalence rates were influenced by OSA diagnostic method and patient recruitment location (community or clinic based). Our findings suggest that OSA may be prevalent in individuals with MCI. Due to the cross-sectional nature of the included studies, we were unable to evaluate a temporal relationship between the conditions (i.e., the occurrence of OSA before or after the development of MCI in patient population). Nevertheless, the clinical impact inferred due to the additive burden of these two disorders demands a closer look into their relationship.

### Population recruitment locations

The studies were conducted in six different countries and enrolled a patient population from a community, general (i.e., public health centers) or specialty neurology clinics that likely contributed to the variations in OSA rates. A clinic-based elderly population is more likely to have individuals with undiagnosed OSA with accompanying major comorbidities that will primarily prompt these patients to seek help. In turn, OSA may remain undiagnosed in this population due to OSA symptoms of, for example [29], memory and concentration being falsely attributed to the aging process or to other disorders by clinicians, hence, transiently decreasing the OSA point prevalence in the clinic-based sample. Alternatively, OSA prevalence rates in MCI are more discernable in a community-based sample and are likely a better representative of the target population. The study utilizing such population reported an OSA prevalence of 27 and 26% in patients with and without MCI, respectively [24]. This rate closely resembled the OSA prevalence (AHI ≥ 5) of 30% estimated in elderly patients between the ages of 50 and 70 years, in a recent epidemiological study [30]. Hence, the high OSA prevalence noted across studies using a clinic-based sample may not be an appropriate representation of the target population (MCI or controls) and may in fact be the result of selection bias.

### Method of OSA diagnosis or screening

The five studies included in this review used different diagnostic methods and criteria to diagnose OSA, which could partially explain the considerable variation in the prevalence rates of OSA. A diagnosis of OSA is made based on an AHI ≥ 5 events/hour for patients reporting symptoms of OSA (e.g. snoring, daytime sleepiness). The prevalence of OSA in MCI for PSG studies using an AHI ≥ 5 was 70 and 71%, respectively [27, 28]. The use of differing definitions for hypopneas in PSG studies has been shown to result in significant variations in the AHI value, which can drastically alter OSA prevalence rates [31]. Both studies, however, had similar apnea and hypopnea definitions according to the American Academy of Sleep Medicine (AASM) guidelines [32]. Diagnostic testing can also be performed using types II-IV portable sleep monitors. One of the five studies used the ApneaLink device and an AHI ≥ 15 events/ hour to diagnose moderate-to-severe OSA demonstrating a prevalence of 27% among the 230 participants with MCI [24]. ApneaLink is a type III portable monitor commonly used for home sleep testing to screen OSA. In adults with moderate-to-high severity of OSA, the ApneaLink has a sensitivity of 75% and specificity of 87% [33]. The specificity of ApneaLink drops to 62% with an AHI cutoff value of 5 that results in mild OSA being undiagnosed [33]. The exclusion of patients with mild OSA in this study population would have resulted in a lower OSA prevalence rate. The Berlin questionnaire [32] is used to screen for high risk patients with OSA and has a pooled sensitivity and specificity of 76 and 45%, respectively, to identify patients with an AHI ≥ 5 events/hour. The study that used the Berlin questionnaire reported a prevalence of 59% among the 138 individuals with MCI [26]. Finally, the study with the lowest prevalence rate employed a patient population with a self-reported OSA diagnosis [2]. The type of OSA diagnostic metric used was not reported. The use of self-reported symptoms would result in significant underestimation of OSA as patients with OSA may be asymptomatic, hence the comparatively low prevalence rate observed in this study. Furthermore, the study used data from the Alzheimer’s disease Neuroimaging Initiative (ADNI) cohort that enrolled only aMCI patients, hence, the associated memory impairments could have partially accounted for the lack in reported information about a previous OSA diagnosis leading to an underestimation of the true prevalence of OSA. Therefore, in memory clinics, a more standardized approach, preferably using objective sleep measurements, needs to be taken when estimating the prevalence of OSA.

### Evidence on association between OSA and MCI

Although several prospective cohort studies [1, 16] have demonstrated that patients with OSA have greater neurocognitive deficits, the risk of OSA and subsequent onset of MCI is seldom explored. In the above mentioned ADNI cohort database, patients with OSA had a younger age onset of MCI by a decade compared to those without OSA, even after adjusting for possible confounding variables [2]. Moreover, continuous positive airway pressure (CPAP) therapy conferred a protective effect, essentially delaying the onset of MCI in those individuals being treated for OSA. Similarly, a number of studies have demonstrated a partial reversibility in cognitive dysfunction with CPAP therapy in individuals with OSA, particularly in the domains of attention, vigilance, executive function and memory [34–36]. Finally, a meta-analysis of cross-sectional studies demonstrated that individuals with AD had a 5-fold risk of OSA compared to healthy age-matched controls [17]. Contrary to this, with the exception of one study [2], there were no significant differences in the risk of OSA among individuals with MCI vs. controls. Perhaps, the additive pathological processes and severity of AD makes these individuals prone to OSA development, which may not be present in those with MCI or early AD (i.e. reverse causation). Nonetheless, the results of these studies signify the importance of early recognition and treatment of OSA in possibly diminishing or delaying the future risk of MCI.

Several mechanisms may contribute to the neurocognitive decline in individuals with OSA, including disturbances in oxidative stress, sympathetic activation, endothelial dysfunction, and systemic and vascular inflammation [37, 38]. Long-standing OSA leads to recurrent intermittent hypoxia and alters sleep architecture, which may lead gradually to brain neurodegenerative processes [39]. A recent review proposed several possible mechanisms linking OSA to dementia, highlighting the important roles chronic sleep architecture impairments may play in neurogenesis, synaptic plasticity and memory consolidation [39]. A neuroimaging meta-analysis assessing the neuro-structural differences between patients with OSA and healthy controls reported significant grey matter reductions in the bilateral parahippocampus, left temporal and right frontal lobes of OSA patients [40]. Whilst there is an adverse impact of OSA on the healthy young brain, and this is greater with the aging middle-aged brain [41], the natural assumption is that the additive burden of OSA may exert greater deleterious effects especially to the elderly brain. Untreated OSA can potentially accentuate the progression of MCI and Alzheimer’s disease [2] in cognitively intact individuals due the accumulation of Alzheimer’s disease biomarkers (amyloid beta and tau proteins) [42, 43], through hypoxic insults and/or disrupted sleep architecture [39].

### Limitations

Our results should be interpreted with caution since this study has some limitations. First, most of the included studies in our review had a small sample size. A small sampling population can lead to an overestimation of the magnitude of an association and ultimately produce high false-positives. Moreover, it may be difficult to interpret the results from studies with a small sample size due to a wider 95% CI that may lead to an imprecise estimate of the effect. Second, we only looked at studies in English language that may have limited our final study count. Third, with a small number of studies and individuals representing this population, difficulties can arise when attempting to conduct a pooled analysis (i.e. meta-analysis), while adjusting for confounding factors, which can lead to unreliable results. Finally, due to the cross-sectional design of the included studies, evaluating a temporal relationship and associations identified are difficult to interpret.

## Conclusions

In summary, the prevalence of OSA in patients with MCI is influenced by OSA diagnostic methods and patient recruitment locations (community or clinic based population). A clinic-based patient population may not appropriately represent general population to estimate OSA prevalence rates. The true OSA prevalence in elderly individuals with MCI may be close to that of the general population with a similar age group, approximately 27%. Longitudinal prospective studies with larger community-based populations and comparable healthy controls, and confirmatory testing are necessary to determine the true prevalence of OSA in MCI. Clinicians caring for patients with OSA and MCI or dementia should consider using standardized methods for diagnosing OSA.

## Additional file

Additional file 1: PRISMA Checklist. (PDF 398 kb)

Additional file 2: Medline (Ovid) Search Terms and Strategy. (DOCX 122 kb)

## Data Availability

All data generated or analyzed during this study are included in this published article.

## Abbreviations

ADNI: Alzheimer’s disease Neuroimaging Initiative
AHI: Apnea Hypopnea Index;
aMCI: Amnestic Mild Cognitive Impairment
APOE: Apolipoprotein E
BMI: body mass index
CI: Confidence Interval
CL: Clinic
COM: Community
CPAP: Continuous Positive Airway Pressure
HNR: Heinz Nixdorf Recall
MCI: Mild Cognitive Impairment
naMCI: Non-amnestic Mild Cognitive Impairment
OD: Oxygen desaturation
OR: Odds Ratio
OSA: Obstructive Sleep Apnea
OSAS: Obstructive Sleep Apnea Syndrome
PSG: Polysomnography
SD: Standard deviation
WHO ICTRP: World Health Organization International Clinical Trials Registry Platform
