# Supplementary material for: The prevalence of obstructive sleep apnea in mild cognitive impairment: a systematic review
Source: BMC Neurol. 2019 Aug 15;19:195. doi: 10.1186/s12883-019-1422-3 (PMC6694482; doi:10.1186/s12883-019-1422-3)
Supplement: Supplementary file 2 — Medline (Ovid) Search Terms and Strategy. (DOCX 122 kb) [file 12883_2019_1422_MOESM2_ESM.docx]

**Table e-1:** Medline (Ovid) Search Terms and Strategy (1946 to May 1, 2018)

| **#** | **Searches** | **Results** |
| --- | --- | --- |
| 1 | exp "Sleep Initiation and Maintenance Disorders"/ | 11471 |
| 2 | exp Sleep Wake Disorders/ | 78331 |
| 3 | exp Sleep/ | 72010 |
| 4 | (sleep* adj2 disturb*).mp. | 13216 |
| 5 | (sleep* adj4 disorder*).mp. | 44254 |
| 6 | (sleep* adj4 character*).mp. | 4016 |
| 7 | (sleep* adj4 impair*).mp. | 2879 |
| 8 | (sleep* adj8 symptom*).mp. | 9967 |
| 9 | (sleep* adj4 syndrom*).mp. | 20716 |
| 10 | (sleep* adj4 issue?).mp. | 426 |
| 11 | (sleep* adj4 problem*).mp. | 6002 |
| 12 | sleepless*.mp. | 664 |
| 13 | insomni???.mp. | 15200 |
| 14 | exp Sleep Apnea Syndromes/ [ MeSH heading changed from exp sleep disordered breathing] | 30969 |
| 15 | exp obesity hypoventilation syndrome/ | 747 |
| 16 | apnea/ and sleep/ [historical] | 263 |
| 17 | apnea/ and (hypoventilation/ or sleep disorders/) [historical] | 213 |
| 18 | (apn?ea adj1 attack*).mp,kw. | 35 |
| 19 | (apn?ea adj1 index*).mp,kw. | 921 |
| 20 | (apn?ea adj1 indices*).mp,kw. | 63 |
| 21 | (apn?ea adj3 monitor*).mp,kw. | 430 |
| 22 | (hypersomni* adj2 periodic breath*).mp,kw. | 11 |
| 23 | (hypersomni* adj2 periodic respirat*).mp,kw. | 8 |
| 24 | (mixed adj2 apnea*).mp,kw. | 184 |
| 25 | (mixed adj2 apnoea*).mp,kw. | 52 |
| 26 | (nocturnal adj2 hypoventilation).mp,kw. | 193 |
| 27 | (nocturnal adj2 hypoxemia).mp,kw. | 375 |
| 28 | (obes* hypoventil* adj2 apne*?).mp,kw. | 30 |
| 29 | (obes* hypoventil* adj2 apnoe*?).mp,kw. | 10 |
| 30 | (obstruct* adj2 hypoapnea*).mp,kw. | 0 |
| 31 | (obstruct* adj2 hypo-apnea*).mp,kw. | 0 |
| 32 | (obstruct* adj2 hypoapnoea*).mp,kw. | 0 |
| 33 | (obstruct* adj2 hypo-apnoea*).mp,kw. | 0 |
| 34 | (obstruct* adj2 hypopnea*).mp,kw. | 339 |
| 35 | (obstruct* adj2 hypopnoea*).mp,kw. | 63 |
| 36 | (pickwick* adj1 syndrom*).mp,kw. | 333 |
| 37 | (sleep adj2 respirat* adj1 disorder*).mp,kw. | 253 |
| 38 | (sleep disorder* adj1 breathing).mp,kw. | 5043 |
| 39 | (sleep disorder* adj1 respirat*).mp,kw. | 110 |
| 40 | (sleep* adj2 hypopn?ea*).mp,kw. | 2010 |
| 41 | apnea-hypopnea*.mp,kw. | 6521 |
| 42 | apneic*.mp,kw. | 2347 |
| 43 | apnoea-hypopnoea*.mp,kw. | 1177 |
| 44 | apnoeic*.mp,kw. | 703 |
| 45 | hypopneic*.mp,kw. | 71 |
| 46 | hypopnoeic*.mp,kw. | 13 |
| 47 | obes* hypoventil* syndrome?.mp,kw. | 930 |
| 48 | osa.tw,kw. | 9063 |
| 49 | osahs.tw,kw. | 1107 |
| 50 | osas.tw,kw. | 3377 |
| 51 | sleep apne*.mp,kw. | 34262 |
| 52 | sleep apnoe*.mp,kw. | 4791 |
| 53 | Polysomnography/ | 18553 |
| 54 | (monitor* adj2 sleep*).mp. | 1260 |
| 55 | polysomnogra*.mp. | 22822 |
| 56 | (PSG and sleep*).mp. | 2940 |
| 57 | somnogra*.mp. | 68 |
| 58 | Berlin questionnaire?.mp. | 322 |
| 59 | STOP questionnaire?.mp. | 30 |
| 60 | STOP-BANG.mp. | 154 |
| 61 | STOPBANG.mp. | 2 |
| 62 | "watch pat 100".mp. | 3 |
| 63 | embletta??.mp. | 41 |
| 64 | or/1-63 [ ~~ OSA or Sleep Disturbances or Sleep Quality ~~ ] | 148639 |
| 65 | Cognitive Dysfunction/ [ new MeSH as of 2017 ] | 8536 |
| 66 | Cognition Disorders/ | 61000 |
| 67 | exp "Mental Status and Dementia Tests"/ [ new MeSH as of 2018 ] | 6402 |
| 68 | (cognitive* adj2 declin*3).mp. | 15371 |
| 69 | (cognitive* adj2 dysfunct*).mp. | 18610 |
| 70 | (cognitive* adj2 disfunct*).mp. | 14 |
| 71 | (cognitive* adj2 impair*).mp. | 48054 |
| 72 | (declin*3 adj2 cognition*).mp. | 380 |
| 73 | (declin* adj2 intellectual* adj2 function*).mp. | 35 |
| 74 | (declin* adj2 mental* adj2 function*).mp. | 37 |
| 75 | (diminish* adj2 cogniti* adj2 function*).mp. | 79 |
| 76 | (diminish* adj2 intellect* adj2 function*).mp. | 7 |
| 77 | (diminish* adj2 mental* adj2 function*).mp. | 3 |
| 78 | (impair* adj2 cogniti* adj2 function*).mp. | 3002 |
| 79 | (impair* adj2 intellect* adj2 function*).mp. | 103 |
| 80 | (impair* adj2 mental* adj2 function*).mp. | 163 |
| 81 | (cogniti* adj2 deteriorat*).mp. | 2072 |
| 82 | (intellect* adj2 deteriorat*).mp. | 453 |
| 83 | (mental* adj2 deteriorat*).mp. | 1374 |
| 84 | (mild* adj2 cogniti* impair*).mp. | 10777 |
| 85 | (mild* adj2 intellect* impair*).mp. | 56 |
| 86 | (mild* adj2 mental* adj2 impair*).mp. | 42 |
| 87 | (mild* adj2 neurocogn* adj2 impair*).mp. | 38 |
| 88 | (mild* adj2 neurocogn* adj2 disorder*).mp. | 79 |
| 89 | (amnestic adj4 impair*).mp. | 1443 |
| 90 | aMCI.ti,ab,kw. [ Amnestic Mild Cognitive Impairment ] | 918 |
| 91 | MCI.ti,ab,kw. and cognit*.mp. [ Mild Cognitive Impairment ] | 6339 |
| 92 | CIND.ti,ab,kw. [ cognitive impairment no dementia ] | 236 |
| 93 | (nonamnestic adj4 impair*).mp. | 47 |
| 94 | exp Neuropsychological Tests/ | 162639 |
| 95 | Alzheimer Disease Assessment Scale-Cognitive-Plus.mp. | 1 |
| 96 | ADAS-Cog Plus.mp. | 2 |
| 97 | ADAS-Cog.mp. | 781 |
| 98 | cognistat.mp. | 65 |
| 99 | dementia rating?.mp. | 2306 |
| 100 | cognitive assessment screening instrument?.mp. | 7 |
| 101 | gpcog.ti,ab,kw. | 14 |
| 102 | general practitioner?? assessment? of cognition.mp. | 15 |
| 103 | mmse.ti,ab,kw. [ mini mental state examination ] | 7775 |
| 104 | mini mental state examination?.mp. | 9740 |
| 105 | minimental state examination?.mp. | 77 |
| 106 | mental status test?.mp. | 94 |
| 107 | "mental status and dementia test?".mp. | 314 |
| 108 | microcog.ti,ab,kw. | 26 |
| 109 | Assessment of Cognitive Functioning.mp. | 111 |
| 110 | mini mental status examination?.mp. | 725 |
| 111 | minimental status examination?.mp. | 6 |
| 112 | mini-cog.ti,ab,kw. | 98 |
| 113 | minicog.ti,ab,kw. | 5 |
| 114 | Montreal cognitive assessment?.mp. | 1101 |
| 115 | cognitive status examination?.mp. | 86 |
| 116 | neurocognitive test*3.mp. | 1178 |
| 117 | Subjective Cognitive Decline Questionnaire?.mp. | 4 |
| 118 | updrs panel?.ti,ab,kw. | 0 |
| 119 | updrs scale?.ti,ab,kw. | 34 |
| 120 | updrs scor???.ti,ab,kw. | 498 |
| 121 | unified parkinson* disease rating?.mp. | 2847 |
| 122 | UPDRS I.ti,ab,kw. | 50 |
| 123 | UPDRS II.ti,ab,kw. | 136 |
| 124 | dementia/ or aids dementia complex/ or alzheimer disease/ or dementia, vascular/ or cadasil/ or dementia, multi-infarct/ or diffuse neurofibrillary tangles with calcification/ or frontotemporal lobar degeneration/ or frontotemporal dementia/ or "pick disease of the brain"/ or primary progressive nonfluent aphasia/ or huntington disease/ or lewy body disease/ | 140451 |
| 125 | dementia rating?.mp. | 2306 |
| 126 | lewy body.mp. | 4706 |
| 127 | (dementia or dementias or demented).mp. | 98102 |
| 128 | (pick? adj2 disease?).mp. | 3894 |
| 129 | Alzheimer*.mp. | 120500 |
| 130 | (pick? adj2 syndrome?).mp. | 57 |
| 131 | parkinson*.mp. | 99525 |
| 132 | or/65-131 [ Mild Cognitive Impairment & related terms ] | 458956 |
| 133 | 64 and 132 [ OSA + MCI ] | 11649 |
| 134 | incidence/ | 229870 |
| 135 | incidence studies/ | 223754 |
| 136 | incidence.mp. | 679904 |
| 137 | prevalence/ | 252216 |
| 138 | prevalence studies/ | 265908 |
| 139 | prevalence.mp. | 540042 |
| 140 | exp risk/ | 1062931 |
| 141 | (risk or risks or risked or risking).mp. | 2005642 |
| 142 | odds ratio/ | 81248 |
| 143 | (odds adj ratio*).mp. | 232626 |
| 144 | or/134-143 [ Incidence or Prevalence or Risk or Odds Ratios ] | 3019947 |
| 145 | 133 and 144 [ OSA + MCI + Prevalence/Incidence/Risk ] | 3593 |
| 146 | limit 145 to english language | 3322 |
| 147 | exp animals/ not (exp animals/ and humans/) | 4456514 |
| 148 | 146 not 147 | 3296 |
| 149 | limit 146 to human | 3295 |
| 150 | 148 or 149 | 3296 |
| 151 | limit 150 to ("all infant (birth to 23 months)" or "all child (0 to 18 years)" or "newborn infant (birth to 1 month)" or "infant (1 to 23 months)" or "preschool child (2 to 5 years)" or "child (6 to 12 years)" or "adolescent (13 to 18 years)") | 581 |
| 152 | 150 not 151 | 2715 |
| 153 | limit 150 to ("all adult (19 plus years)" or "young adult (19 to 24 years)" or "adult (19 to 44 years)" or "young adult and adult (19-24 and 19-44)" or "middle age (45 to 64 years)" or "middle aged (45 plus years)" or "all aged (65 and over)" or "aged (80 and over)") | 2462 |
| 154 | 152 or 153 | 3056 |
| 155 | remove duplicates from 154 [ OSA + MCI + Prevalence/Incidence/Risk + Limits ] | 3015 |
